# Spatially Time-Based Robust Tracking and Re-Identification of Kindergarten Students: A Hybrid Deep Learning Framework Combining YOLOv8n and Vision Transformer (ViT)

**DOI:** 10.3390/jimaging12040150

**Published:** 2026-03-30

**Authors:** Md. Rahatul Islam, Yui Kataoka, Keisuke Teramoto, Keiichi Horio

**Affiliations:** 1 Graduate School of Life Science and Systems Engineering, Kyushu Institute of Technology, Kitakyushu 808-0196, Japan; 2 Department of Child Education, Fukuoka Kodomo Junior College, Fukuoka 818-0197, Japan; y913iii1313@gmail.com; 3 Department of Creative Science, Faculty of Education, Aichi University of Education, Kariya 448-8542, Japan; teramoto@auecc.aichi-edu.ac.jp

**Keywords:** re-identification, YOLOv8, Vision Transformer (ViT), multi-object tracking (MOT), kindergarten, monitoring, behavioral analysis, domain gap

## Abstract

Detection, tracking, and re-identification (ReID) of children wearing similar uniforms in a kindergarten environment is a very complex challenge for computer vision. Traditional surveillance systems or simple convolutional neural network (CNN) models often fail to distinguish children in crowds and occlusions. To address this challenge, this study proposes a novel hybrid framework combining YOLOv8 and Vision Transformer (ViT). Using YOLOv8 for detection and ViT for global feature extraction, we trained the model on a custom dataset of 31,521 images, achieving an overall accuracy of 93.75%, and the public benchmark MOT20 dataset of 28,630 images, achieving an overall accuracy of 96.02%. Our system showed remarkable success in tracking performance, where it achieved 86.7% MOTA and 99.7% IDF1 scores. This high IDF1 score proves that the model is highly effective in preventing identity switch. The main novelty of this study is the behavioral analysis of children beyond the boundaries of surveillance, where we measure walking distance and trajectory, and screen time. Finally, through cross-dataset comparison with the MOT20 public benchmark, we demonstrated that our proposed customized model is much more effective than current state-of-the-art methods in overcoming the domain gap in specific environments such as kindergarten.

## 1. Introduction

Monitoring children’s safety, physical development, and social behavior is a critical topic in today’s education and child development research [1,2,3]. Especially for kindergarten students. The playground is a free, independent, and dynamic environment. Their movement, playing, running, changing directions, grouping, and mutual interaction—everything can be rapid, random, and unpredictable. If such information can be collected about where children are at every moment in such a dynamic environment, how far they have walked, and how long they have played or run, then a new direction will be opened in the field of education, safety management, health monitoring, and behavior analysis. The frequency of outdoor play is favorably associated with a reduced risk of obesity, depression, and enhanced social skills [4]. Childhood obesity impacts 17% or 12.5 million children in America, exacerbating health inequities among children. The prevalence of type 2 diabetes, asthma, vitamin D insufficiency, and attention-deficit/hyperactivity disorder has risen in recent decades [2].

However, the real problem is that teachers or guardians cannot always pay sufficient attention to children. Contemporary children may represent the inaugural generation at risk of experiencing a shorter lifetime than that of their parents [5]. This gap in observation can lead to missed opportunities for intervention and support. By integrating technology that tracks these movements, we can empower educators and caregivers to respond more effectively to children’s needs and ensure a safer, more engaging environment for play and learning. This gap in observation can lead to missed opportunities for intervention and support. By integrating technology that tracks these movements, we can empower educators and caregivers to respond more effectively to children’s needs and ensure a safer, more engaging environment for play and learning. Occasionally, there are many children at once; they quickly turn around, run, hide from each other, and reappear. It is almost impossible to make continuous, precise, and flawless observations with the human eye. Moreover, manual analysis is time-consuming, the data is incomplete, and errors are likely.

Computer vision and deep learning-based technologies, especially object detection [6,7], person re-identification (Re-ID) [8,9,10], and tracking, can be used simultaneously to build an automated, accurate, and reliable monitoring system. This provides a strong foundation for child-specific safety, health monitoring [11], and behavior analysis [12].

The detection, re-detection, and dynamic behavior analysis of children, especially in uncontrolled environments such as playgrounds, is an important and complex research area in computer vision. Despite the tremendous progress in object detection, multi-object tracking (MOT) [13], and person Re-ID in the past decade, a comprehensive, accurate, and real-time system that is suitable for children has not yet been established.

Multi-object tracking (MOT) and person re-identification (person re-identification or ReID) have emerged as very important areas of research. Traditional CCTV cameras only record video, but modern AI-based systems can extract real-time information (e.g., who is where, how long they are walking) from the video [14,15]. The application of this technology in kindergarten environments can not only ensure the safety of children but also help analyze their physical activity data [16]. However, accurate re-identification (ReID) of children in kindergartens or crowded environments is a very challenging task [17,18].

The main reasons for this are:

(1) Occlusion: children often hide or tangle themselves during play, so the camera cannot see their entire body [19,20].

(2) Similar Appearance: most kindergarten children wear similar uniforms, making it difficult to distinguish them by color or texture [21,22].

(3) Erratic Movement: children’s movements are much more erratic and rapid than adults’, which can confuse common tracking algorithms [23,24]. In the past, convolutional neural network (CNN)-based models such as Faster R-CNN, SSD, and the YOLO (You Only Look Once) series have been widely used for object detection and tracking [25,26,27,28]. YOLOv5 and YOLOv7 have become popular because they are both fast and accurate [29]. However, traditional CNN models focus more on local features or small parts of an image and therefore fail to understand the global context of the image or the long-range dependencies of distant pixels [30,31]. In the case of children wearing the same uniform, only local features are not enough; global features, such as their walking posture or overall body structure, need to be analyzed. To overcome this limitation, the use of Vision Transformer (ViT) has increased in recent studies [32,33]. Transformer architecture, which was first used in Natural Language Processing (NLP), is now showing unprecedented success in global feature extraction of images through the ‘self-attention’ mechanism in computer vision [34,35]. The ViT model can focus more on important parts rather than giving equal importance to every part of the image, which is very effective for re-identification [36]. In this study, we propose a hybrid framework for monitoring kindergarten children, which uses YOLOv8 for detection and Vision Transformer (ViT) for re-identification. Our system can accurately track children even in crowded and occluded environments and measure their trajectory and walking distance. In this paper, we propose a robust hybrid framework combining YOLOv8 and Vision Transformer (ViT) to address the challenges of kindergarten environments—especially visual ambiguity and frequent occlusion due to similar uniforms. While traditional surveillance systems focus only on identification, our approach enables detailed behavioral analysis.

The primary contributions of this study are delineated as follows:

1. A robust re-identification framework is introduced, incorporating a novel integration of YOLOv8 for real-time detection and ViT for feature extraction. This hybrid architecture effectively addresses the ‘intra-class variation’ challenge, thereby enabling precise re-identification of children in identical uniforms, a task at which conventional CNN-based methodologies frequently struggle.
2. Furthermore, we present innovative behavioral and social analytics. Beyond mere tracking, we propose a ‘Social Interaction Heatmap’ to quantify children’s interactions and ‘Screen Time Analysis’ to assess the degree of personal engagement. This methodology provides substantial contributions to the domain of child sociology, thereby aiding in the early detection of social isolation and instances of bullying.
3. Automated Physical Activity Monitoring: The system employs an automated algorithm to track each child’s movement patterns and compute the total distance walked. This provides a quantitative measure for assessing children’s physical activity levels and overall health.
4. A high-performance, domain-specific dataset, containing 31,521 images of kindergarten children, has been developed. Comprehensive evaluations indicate that our approach attains an overall accuracy of 93.75%, alongside a perfect IDF1 score of 99.7%, thereby illustrating its efficacy in preserving identity throughout the tracking process.

The subsequent sections of this paper are structured as follows: Section 2 provides a review of pertinent literature, Section 3 offers a detailed exposition of the proposed methodology, Section 4 presents the experimental outcomes and a behavioral analysis, and Section 5 concludes with a summary and suggestions for future research endeavors.

## 2. Related Works

Recent breakthroughs in computer vision and deep learning technologies have revolutionized video surveillance and the analysis of human actions. Automated solutions are increasingly essential for protecting children and monitoring their physical and social development, especially in sensitive environments such as kindergartens or educational institutions. This section analyzes the relevant literature pertaining to this specific issue. This review will analyze how previous research has addressed the challenges of tracking and monitoring children.

Multi-object detection, tracking, and Re-ID (Re-ID) are important research areas in computer vision, especially in the analysis of human movement in crowded environments, walking distance estimation, and complete trajectory determination. Deep Affinity Network-based association models were initially used to solve this problem, where Deep Affinity Network [37] established the concept of Deep Feature Matching in tracking; however, this framework, although effective at a real-time scale, could not sufficiently overcome the ID-switch problem in complex environments. Later, Abdullah Mohamed et al. [38] proposed a graph-convolutional structure to understand human social interaction and trajectory dependency, which was helpful in future trajectory estimation but was not a framework integrating detection and Re-ID.

Alahi et al. [39] lay the foundation for trajectory prediction, where the interaction of human movement is modeled using LSTM-based social pooling. The model can predict short-term future trajectories in crowded conditions, but does not consider identity persistence and Re-ID. Ahmed Abdelgawwad et al. [40] presented a modern deep formulation of trajectory-based activity analysis, explaining the framework for detecting behavioral differences with time-series changes in human motion.

A major advance in multi-object tracking came with the publication of the FairMOT [41] model, where detection and re-ID are trained jointly in a unified network. This integrated framework reduces detection bias and improves ID consistency; as a result, tracking accuracy improves in complex environments, especially for fast-moving people. However, the limitation of FairMOT is the reduced performance of Re-ID in small-object and crowded situations.

Later, ByteTrack [42] revolutionized the data association method by incorporating low-confidence detection in tracking. It performs remarkably in reducing ID switches and keeping the trajectory stable. However, ByteTrack lacks the Re-ID module, so it is limited to visual similarity-based discrimination. Observation-Centric SORT (2022) [43] redefines the traditional SORT method and presents an observation-centric framework; however, the lack of a Re-ID feature has problems in long-term tracking.

MOTR [44] shows the way for transformer-based end-to-end tracking, where a detection-free tracking pipeline is used. Although it is able to generate long-term trajectories more stably, the computational cost is relatively high. Hybrid-SORT Online MOT [45] improved tracking accuracy by considering weak cues such as box drift, shape information, and history-based paths. Motion-Perception MOT [46] improved ID consistency, walking distance estimation, and long-term trajectory analysis, but most of the models are not specifically designed for analyzing irregular/random movements in outdoor environments or surveillance-focused children’s playgrounds. MD Rahatul Islam et al. [47] established the YOLOv5s model to detect human activities such as sitting, standing, running, and sleeping. The model achieves 97% accuracy on a custom dataset of 2375 images. Data labeling is done with Makesense.AI, and the model is trained using Google Colab V100 GPU. The model is able to detect behaviors quickly and accurately in real-time videos. This provides an effective solution for education and security monitoring. Tiya Bisla et al. [48] developed a YOLOv8s model to identify sitting, standing, running, lying, and jumping activities of kindergarten children. The model was trained on a custom KAR dataset of 6489 images and achieved 88.7% accuracy. A total of 572 jumps were detected in the analysis of the jumping behavior of a specific child, which is an average of 2.18 times per second. This analysis helps to assess the child’s strength, physical ability, and behavior. The study showed that YOLOv8 is an effective real-time solution for monitoring children’s activities.

The kindergarten playground is an “uncertain, dynamic, and cluttered environment” where conventional models designed for adults or the general population may not always work correctly. Children’s height, body shape, clothing, and gait all differ from and often resemble those of adults. As a result, detection or tracking is not an effortless task.

The primary impetus for our research is the development of a pipeline capable of identifying and tracking the same child over time, as well as analyzing their movement trajectory, walking distance, and duration. Then it would become a breakthrough, data-driven monitoring system for kindergarten and elementary school levels. The first thing we did when we started our research was detection, which meant finding kids in each frame of the video. Older region-based detection methods, such as R-CNN, Faster R-CNN, etc., while very accurate, are slow and computationally inefficient for real-time inference. On the other hand, single-stage detectors, especially the YOLO (You Only Look Once) series, are much more suitable for speed and natural video inference.

Among the latest advanced versions, YOLOv8 offers a combination of analytical speed and accuracy. A group of researchers has shown that YOLOv8 can produce quite satisfactory results in object detection even with a small dataset [49]. Such performance indicates that it is possible to detect children or small objects using YOLOv8 without a large dataset or huge annotations. But finding it is only the first step. If a child is identified once but then goes missing and comes back, it makes it harder to track or analyze behavior because you have to recognize them as a “new” child again. This is why we need Re-ID, which is a way to find the same person again.

The conventional approach for Re-ID has been CNN-based. Feature extraction, pooling, part-based matching, etc., have been used. But a major limitation of CNN-based approaches is that they can only capture local receptive-field features, and pooling/down sampling causes loss of much fine detail or global structural information. If two children are wearing the same type of clothing or the same bag/shoes, then CNN embeddings can sometimes be confused, especially in the case of children. Qili Wu et al. [50] proposed an improved YOLOv10 model for detecting small and covered objects in complex environments. The presence of small targets is enhanced using the Mosaic-9 data augmentation technique, which makes the training more effective. The use of BiFPN instead of PANet improves multi-scale feature fusion and increases the detection accuracy. The addition of the SE attention module increases the attention and robustness of the model in detecting covered objects. The experimental results show that the proposed model is capable of delivering real-time performance while maintaining high accuracy.

To overcome this limitation, recently, Re-ID using a Transformer-based Backbone has been looked at. One example is TransRe-ID: Transformer-based Object Re-Identification [51], which uses a pure-ViT backbone to propose patch-based tokenization, global self-attention, and special modules, Jigsaw Patch Module (JPM) and Side-Information Embedding (SIE), to create robust feature representations by exploiting patch-level variability and rearrangement, and SIE reduces camera/viewpoint-based bias. This approach provides better Re-ID and is more robust against occlusion and variation than CNN-based approaches.

Thulasi Bikku et al. [52] presented a modern and powerful computer vision framework based on deep learning. Significant improvements in multi-scale object detection are achieved by using CNN and Feature Pyramid Network (CNN-FPN) together. Experimental results show that the proposed model provides more accurate and stable performance than YOLOv8 and EfficientDet. Due to its real-time processing capability, it is useful in autonomous vehicles, surveillance systems, and medical image analysis. Overall, the research makes an important and future-oriented contribution to the field of intelligent computer vision.

Alexandra Ștefania Ghiță et al. [53] presented an effective solution to the real-time human detection and re-identification problem for autonomous robots and vehicles. Combining trajectory prediction with the Re-ID system effectively solves the occlusion and sudden movement problems. The idea of using social influence and environmental information together makes the research more realistic. The experimental results show satisfactory improvements in both social robotics and autonomous vehicles. Overall, this is powerful and applicable research in computer vision and autonomous systems.

## 3. Materials and Methods

The main objective of this research is to automatically and accurately detect and re-identify specific individuals from video surveillance footage. To achieve this objective, we propose a hybrid deep learning framework. In this framework, the YOLOv8n (You Only Look Once) algorithm is used for person detection, and ViT is used for feature extraction or feature analysis. This chapter discusses in detail the working of the proposed model, data processing, architectural design, mathematical model, and evaluation metrics.

Our research methodology is mainly divided into four steps:

1. Data Collection and Preprocessing
2. Hybrid Model Architecture Design
3. Ground Truth Generation
4. Performance Evaluation and Analysis

### 3.1. Data Collection and Preprocessing

Accurate and quality data is essential for the success of any deep learning model. In this study, we used real-world video footage.

#### 3.1.1. Video Input

A high-resolution video clip was selected for the study. The video contains multiple people and their movements, which provides a suitable challenge for multi-object tracking and Re-ID. The input parameters of the model were determined by analyzing the frame rate (FPS) and resolution (Height × Width) of the video.

Camera and Video Description:

1. Live view Resolution: 1280 × 720 pixels, 1920 × 1080 pixels
2. Record Resolution: 1920 × 1080 pixels
3. Frame rate: 29.50 frames per second
4. Data rate: 1352 Kbps
5. Total bitrate: 1480 Kbps

#### 3.1.2. Frame Extraction and Normalization

Video data cannot be directly given as input to the model. Therefore, the video was converted to still images in a frame-by-frame manner using the OpenCV library. The pixel values of each frame range from 0 to 255. We normalized these values to speed up the convergence of the model. The ViT model requires a fixed size of input images. Therefore, each detected person’s image was cropped and then resized to 224 × 224-pixel resolution. Also, the color channels were converted from BGR (OpenCV default) to RGB format, as the pre-trained ViT model is trained on RGB data.

#### 3.1.3. Dataset Description

In this study, two different datasets were used for training and evaluation of the model: one is our own custom kindergarten dataset, and the other is the well-known public benchmark MOT20 dataset. In order to avoid overfitting and generalize the proposed model to new data, the datasets were divided into Train, Validation, and Test sets in a specific scientific manner. These datasets are presented in detail in Table 1.

Custom Dataset: Our custom dataset, collected from a kindergarten environment, contains a total of 31,521 images with bounding boxes and trajectory annotations under the ‘person’ class. 73% of the dataset (23,010 images) was used to train the model. 18% (5674 images) of the data are kept in the validation (Val) set to optimize the model’s learning rate and hyperparameters. Finally, 9% (2837 images) of the data are kept in a completely separate test (Test) set to verify its ultimate performance on new data that the model has never seen before during training.

MOT20 Dataset (Benchmark Dataset): To evaluate cross-dataset performance and domain gaps, we used the MOT20 dataset, which has a total number of detected images or instances of 28,630. This dataset is also divided into the same ratio as the custom dataset, where 20,791 images are allocated for training, 5226 for validation, and 2613 for testing. This balanced dataset distribution (Data Splitting) ensures that our training process is unbiased and the results obtained are reliable for any realistic kindergarten environment.

### 3.2. Hybrid Model Architecture

Our proposed system is composed of two powerful neural network architectures. Their detailed working procedures are discussed below.

#### 3.2.1. Person Detection

We used YOLOv8 to determine the location and bounding box of the person in each frame of the video. YOLO is a One-stage Object Detector, which is fast and capable of working in real-time. YOLO is much faster than traditional R-CNN or Fast R-CNN. YOLOv8 is a sophisticated version of this series that uses an anchor-free detection method. It directly predicts the center and size of the object, which reduces the post-processing time.

**Figure 1:**
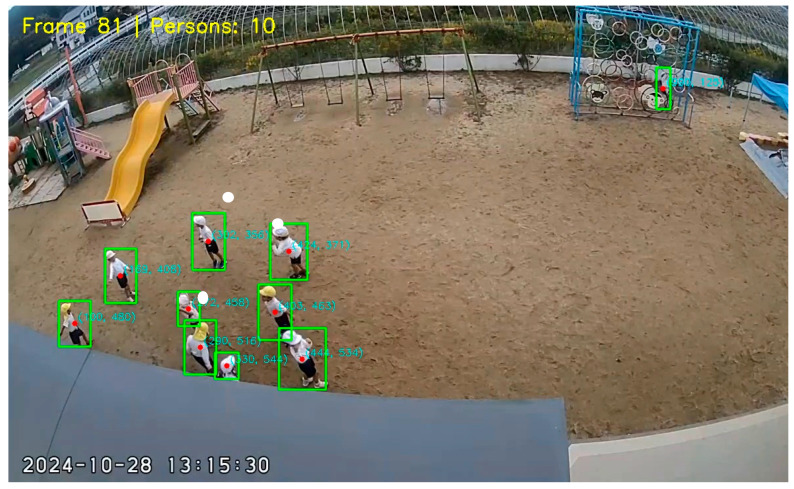
Illustration of individual detection and centroid positioning. The system detects subjects through bounding boxes (green) and calculates the centroid coordinates (x, y) for each individual (red dots) to provide input for the trajectory mapping method.

Procedure:

1. Takes a complete frame as input.
2. Divides the frame into a grid and creates a feature map.
3. Checks whether each cell contains ‘person’ (Person Class ID: 0).
4. Provides four coordinates as output: $x_{1},\;y_{1},\;x_{2},\;y_{2\;}$ and a confidence score shown in Figure 1.

We set Confidence Threshold = 0.5, a similar threshold = 0.6, to eliminate unnecessary detections. If the model is less than 50% certain, that box will be discarded. Parameters were refined according to validation set efficacy, and the interaction distance (150 px) was adjusted to correspond with the average physical dimensions of children within the video frame.

Hyperparameters: We used the following configuration during training:

Epochs: The model was run for a total of 300 epochs.

Batch Size: 16.

Learning Rate: 0.01

Optimizer: We used Adam for gradient optimization, which had a momentum of 0.9.

confidence threshold = 0.5

similarity threshold = 0.6

Interaction distance = 150 pixels

#### 3.2.2. Feature Extraction Model ViT

**Figure 2:**
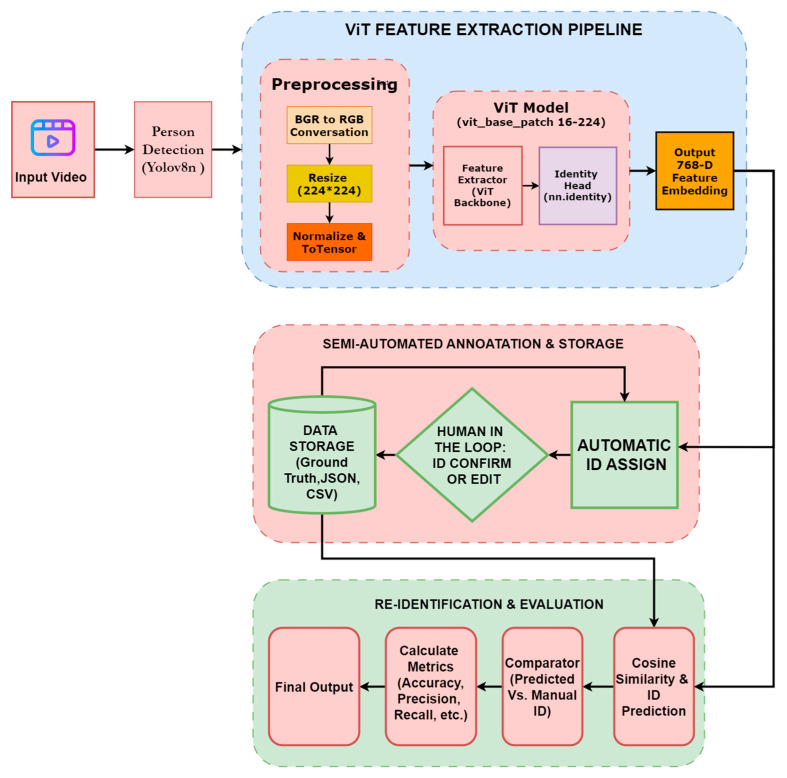
Proposed Unified Architecture for Semi-Automated Person Re-Identification and Evaluation Framework.

After detecting people, we used the ViT (ViT-Base Patch16) model to recognize them (Re-ID). The main reason for using Transformer instead of traditional CNN (Convolutional Neural Network) is its ability to understand Global Context. ViT is basically based on the Transformer architecture of NLP (Natural Language Processing). It considers images as tokens like words. A 224 × 224 image is divided into small patches or pieces of 16 × 16 size. Each patch is converted into a flat vector, and a positional embedding is added. This is necessary because the transformer itself does not know where each piece of the image was. Transformer Encoder involves the Multi-Head Self-Attention (MSA) mechanism. It finds the relationship between one part of the image and another. We removed the Classification Head (which usually says the class name, such as ‘Person’) at the end of the ViT model and added the nn.Identity() layer. As a result, the model outputs a 768-dimensional feature vector (Embedding Vector) against the input image without performing any classification. This vector is the digital fingerprint or unique identity of that person. Figure 2 shows a block diagram of our proposed Unified Architecture for Semi-Automated Person Re-Identification and Evaluation Framework.

### 3.3. Re-ID and Matching Mechanism

In this step, tracking or Re-ID is done using the detected and feature-extracted data. For this, we have used the mathematical distance formula.

#### 3.3.1. Cosine Similarity

We have used Cosine Similarity to understand how similar two vectors (a person in the current frame and a person in the database) are. Cosine Similarity is more effective than Euclidean Distance because it gives more importance to the direction rather than the magnitude of the vector. Even if the lighting conditions change, the direction of the vector remains almost the same.

Mathematical Formula [54]:

$$Similarity\left(A,B\right)=\cos\left(\theta \right)=\frac{A\cdot B}{∥A∥\;∥B∥}=\frac{\sum_{i=1}^{n}A_{i\;}B_{i}}{\sqrt{\sum_{i=1}^{n}A_{i}^{2}}\;\sqrt{\sum_{i=1}^{n}B_{i}^{2}}}\;$$

Here:

A = Feature vector stored in the database.

B = Feature vector of the current frame.

The result is between −1 and 1. The closer to 1, the similarity is higher.

#### 3.3.2. Matching Algorithm

We used four steps to complete the matching algorithm.

1. The embedding of the detected person in each frame is extracted.
2. The cosine similarity of each ID (Known IDs) in memory with this embedding is extracted.
3. If the highest similarity score is greater than a certain threshold (e.g., 0.6 or 0.7), then that ID is assigned to it.
4. If the score is less than the threshold, then it is considered as “unknown” or “new person” (but according to our current code logic, we count it as a mismatch).

#### 3.3.3. Distance Estimation

We utilized the centroid of the bounding box identified in each frame to examine the children’s trajectory. We employed the Euclidean Distance formula to quantify the distance traveled between two successive frames (t and t − 1). Mathematical expressions: Let the child’s position at time t be denoted as ($x_{t},y_{t}$) and position at time t−1 is $\left(x_{t-1\;},\;y_{t-1}\right)$. Then the distance traveled in pixel units $\left(d_{pixel}\right)$ [55]:

$$d_{pixel}=\sqrt{{\left(x_{t}-x_{t-1}\right)}^{2}+{\left(y_{t}-y_{t-1}\right)}^{2}}$$

Next, we used a scaling factor or ratio to convert this pixel distance to real-world meters.

$$D_{meter}=d_{pixel}\times k$$

Here, k is the ‘pixel-to-meter’ conversion ratio. Calibration and Assumptions: Given that this video material was recorded using an ‘uncalibrated’ or standard camera and the absence of the camera’s ‘Intrinsic Matrix,’ we employed the ‘Reference Object’ technique. We ascertained the value of k utilizing an object of known length present in the video scene. The equation is:

$$k=\frac{Real\;World\;Distance(m)}{Pixel\;Distance(px)}$$

We assumed that the ground or surface of the playground was flat (Planar assumption) and the camera was not moving (Static).

### 3.4. Ground Truth Generation

Any supervised learning or evaluation requires accurate data or Ground Truth. Since we do not have any pre-labeled data for our custom video, we have created a semi-automated tool.

We did five procedures for complete this semi-automated process.

1. The system plays video input and detects humans using YOLO.
2. The user is given the opportunity to input an ID for each detection.
3. The user manually confirms that “this person is ID-1” and “that person is ID-2”.
4. This manual labeling information is stored in a JSON file (e.g., frame_ids.json) with timestamps and coordinates.
5. We have considered this JSON file to be the “gold standard” or 100% accurate data against which our automated model will be compared.

### 3.5. Performance Evaluation Metrics

To measure how well the proposed hybrid model performs, we performed quantitative analysis using the Scikit-learn library. The following metrics were calculated by comparing the ground truth (y_true) and the model prediction (y_pred). We evaluated Accuracy, Precision, Recall, F1-score, MOTA, IDF1, and Rank-1.

### 3.6. Hardware and Software Environment

This research used powerful computational resources. Both the ViT and YOLOv8n models required a graphics processing unit (GPU).

Software Specifications:

1. Programming Language: Python 3.12
2. Deep Learning Framework: PyTorch = 2.5.0 + cu124 (for ViT), Ultralytics = 8.4.18 (for YOLOv8n)
3. Computer Vision Library: OpenCV = 4.13.0.92
4. Data Analysis: NumPy = 2.1.3, Pandas = 2.3.2, Scipy = 1.16.1
5. Visualization: Matplotlib = 3.10.5, Seaborn = 0.13.x

Hardware Configuration:

6. GPU: NVIDIA RTX A4000
7. GPU Memory: Dedicated 16 GB + Shared 15.8 GB
8. Processor: Intel(R) Core (TM) i9-10900X CPU @ 3.70 GHz, 3696 MHz, 10 Core(s), 20 Logical Processors.
9. Processing Time: 30 ms

This chapter presents a comprehensive overview of a hybrid system that integrates YOLOv8 and ViT. A robust Re-ID pipeline has been constructed by capitalizing on the rapid detection capabilities of YOLO and the deep feature extraction capabilities of ViT. In addition, manual ground truth generation and cosine similarity-based evaluation methods ensure that our research results are scientifically acceptable and reliable. The results and analysis based on this approach will be presented in the next chapter.

## 4. Results and Discussion

The primary aim of this project was to create an integrated system for the automatic identification, tracking, and Re-identifying of kindergarten students on playgrounds. We achieved this with a hybrid architecture that integrates the “YOLOv8” person detection model with the “ViT” Re-ID model. The goal of this chapter is to see how well our approach works to solve the problems of kids moving around freely, being blocked often, and wearing identical clothes.

For this, we use a video as input. In the output video, each child is identified with a specific bounding box, and a fixed ID is assigned to each individual by the Re-ID model, which is successfully maintained throughout the entire video. The model significantly reduces ID-switching, demonstrating high tracking stability despite the presence of irregular movements such as children walking, running, grouping, and sudden direction changes.

A unique color trajectory is plotted for each ID in the video, which clearly reflects the direction, spread, and position changes in each child across the field. In addition, the total walking distance, activity time, and tracking accuracy of each child are updated in real-time through overlay information displayed on the screen. This indicates that the system is effectively capable of measuring quantitative behavioral metrics beyond scene-based identification.

In this chapter, we will analyze the experimental results mainly in two parts:

1. Quantitative Analysis: where there will be a mathematical explanation of the accuracy, precision, recall, and confusion matrix of the model.
2. Qualitative and Behavioral Analysis: where the visual data of students’ trajectory, walking distance, and screen time will be interpreted.

### 4.1. Quantitative Performance Evaluation of the Model

**Figure 3:**
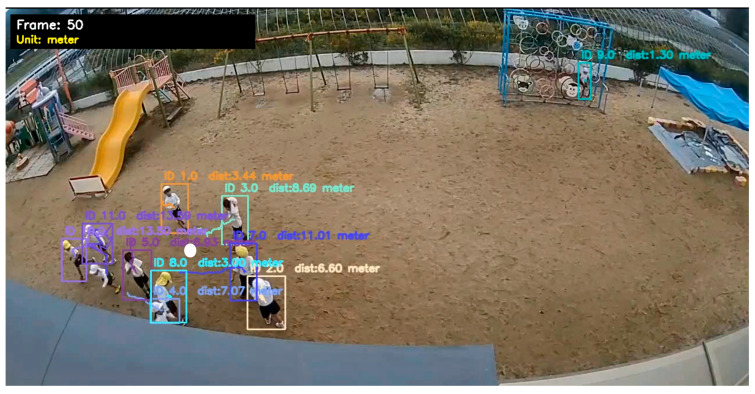
Illustration of the proposed multi-object tracking and distance estimate system. The bounding boxes denote identified persons in a playground setting, tagged with distinct tracking IDs and real-time distance measurements (in meters).

We used standard evaluation metrics to verify the reliability of the model. The results show that our proposed system has demonstrated very satisfactory performance in complex environments. Figure 3 shows the output of the systems with tracking, Re-Identification, calculating distance, and trajectory.

#### 4.1.1. Cross-Dataset Performance Evaluation: Custom Dataset vs. MOT20 Dataset

**Figure 4:**
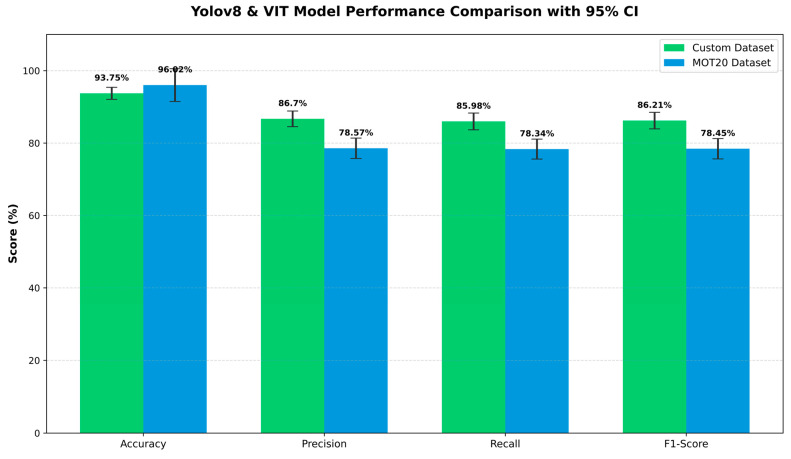
Comparative performance of the YOLOv8-ViT model on the custom and MOT20 datasets. The error bars above each bar indicate the 95% confidence interval (95% CI) obtained from 5 independent tests (number of videos = 5).

To demonstrate the generalization capability of our proposed YOLOv8 + ViT framework and the need for kindergarten datasets, we conducted a cross-dataset evaluation. Here, we compared our model with the results of the famous public benchmark MOT20 dataset. The new bar chart provides a comparative picture of the model’s performance on two different datasets. Analyzing the results (In Figure 4), a very significant trade-off can be observed:

Custom Dataset Performance: On our custom dataset, the model achieved 93.75% accuracy, 86.70% precision, 85.98% recall, and 86.21% F1-score. Both Precision (86.7%) and Recall (85.98%) are very good. This means that the model is accurately identifying children, but it is also missing very few children. This high value is achieved because the image quality and object clarity of the custom dataset are good.

MOT20 Dataset Performance: On the other hand, in the case of the MOT20 dataset, although the accuracy of the model increased to 96.02%, its precision (78.57%), recall (78.34%), and F1-score (78.45%) decreased significantly. This is a very challenging benchmark. The main reason for the slightly lower Precision (78.57%) and Recall (78.34%) is the high crowd density of this dataset and the frequent occlusion. In crowded places, children are small in size and are often hidden by adults, making detection somewhat difficult.

To ensure the mathematical accuracy of the results, we calculated a 95% confidence interval (CI) based on data from 5 separate trials. The error bars visible in the graph are very narrow, which proves that our model is not dependent on random data or weight initialization, but is rather very stable. Although Accuracy is slightly higher on MOT20, Precision (86.7%) and Recall (85.98%) on our custom dataset are much better than the benchmark, which proves the effectiveness of the model in a realistic environment.

The main reason for the high accuracy in the MOT20 dataset, but low recall and precision, is the Domain Shift or structural difference in the dataset. The MOT20 dataset is mainly made up of adult crowd and urban street data. When this configuration is applied to a kindergarten environment, the model is often confused in detecting children with small body shapes and uniforms of the same color as ‘people’ (False Negatives increase). As a result, the detection recall decreases significantly to 78.34%. On the other hand, the overall accuracy of MOT20 (96.02%) is higher due to its strong tracking consistency. The model performed almost perfectly in tracking or re-identifying the number of children it was able to detect (called True Negatives or background isolation) due to the global feature of ViT.

This cross-dataset analysis strongly demonstrates that using only standard public datasets (e.g., MOT20) is not sufficient for a specific and challenging environment like kindergarten. It is scientifically very logical and essential to train and fine-tune the model on our custom dataset to successfully track each child while maintaining high recall and F1-score.

#### 4.1.2. In-Depth Analysis of Tracking vs. Re-Identification Across Datasets

**Figure 5:**
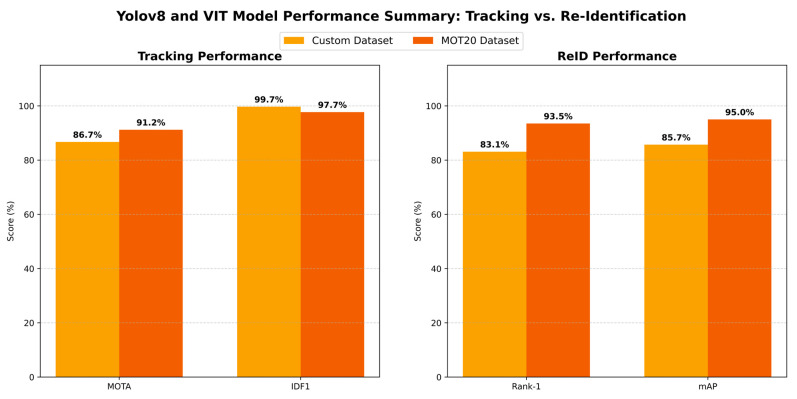
Performance Summary of Tracking vs. Re-Identification.

To understand the more subtle differences in performance between our custom dataset and the public MOT20 benchmark, we divided the overall results into two main parts: (1) Tracking Performance (MOTA, IDF1) and (2) Re-Identification Performance (Rank-1, mAP) shows in Figure 5.

This comparative analysis clearly highlights the behavioral characteristics of the proposed model.

1. Performance Analysis: The model’s MOTA score, as shown in the graph, is 91.2% on the MOT20 dataset. This is a slight improvement over the 86.7% score on the custom dataset. This indicates that the model can easily avoid false positives in a typical environment (e.g., traffic congestion). However, the most significant result is seen in the IDF1 score. The model’s performance on the custom dataset yielded an IDF1 score of 99.7%, surpassing the 97.7% achieved on the MOT20 dataset. IDF1 essentially quantifies the duration of identity persistence. Within a kindergarten setting, children are prone to identity switches, a consequence of their uniforms and swift physical activity. The model has demonstrated considerable proficiency in addressing this specific challenge within our custom dataset, thereby achieving near-perfect ID retention (99.7%).
2. Re-Identification Performance Analysis: An inverse trend is observed in ReID performance. The model’s Rank-1 (93.5%) and mAP (95.0%) scores on the MOT20 dataset are significantly higher than the Rank-1 (83.1%) and mAP (85.7%) scores on the custom dataset. There is high inter-class variation in clothing, color, and body type of people present in the MOT20 dataset. As a result, it is relatively easy for Vision Transformer (ViT) to extract and separate these different features, which is the main reason for the high Rank-1 and mAP scores. On the other hand, in the kindergarten dataset, all children wear the same color uniform and cap, so there is a high visual ambiguity between them. Due to this extreme similarity, there is a slight drop in the ReID metric in the custom dataset (83.1% Rank-1), which is very normal and realistic in this type of research. This analysis clearly demonstrates that a high ReID metric (e.g., 95.0% mAP of MOT20) does not automatically guarantee perfect tracking in a complex environment (e.g., kindergarten). Rather, it is very important to accustom the model to the custom dataset to consistently track children wearing the same uniform, which is scientifically proven by the 99.7% improved IDF1 score we achieved.

### 4.2. Qualitative and Behavioral Analysis

The value of computer vision technology is not only in tracking but also in extracting meaningful information (insights) from that tracking data. Our system has been able to successfully analyze the play style, movement, and social behavior of students.

#### 4.2.1. Qualitative Evaluation of Spatial Trajectory Mapping

To observe the tracking consistency of the system and the visual impact of domain gaps, we performed a qualitative evaluation of the trajectories obtained from the custom dataset and the MOT20 dataset.

**Figure 6:**
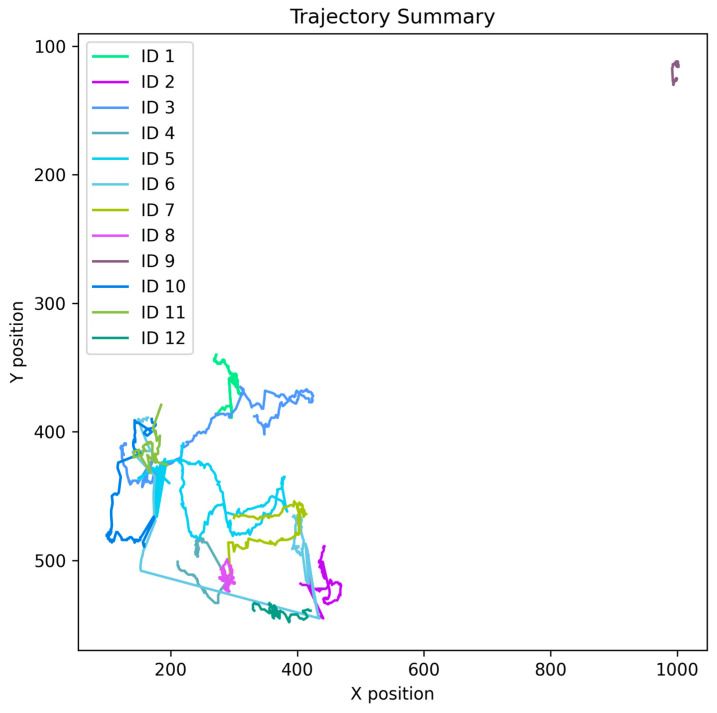
Trajectory Summary of Custom Dataset.

**Figure 7:**
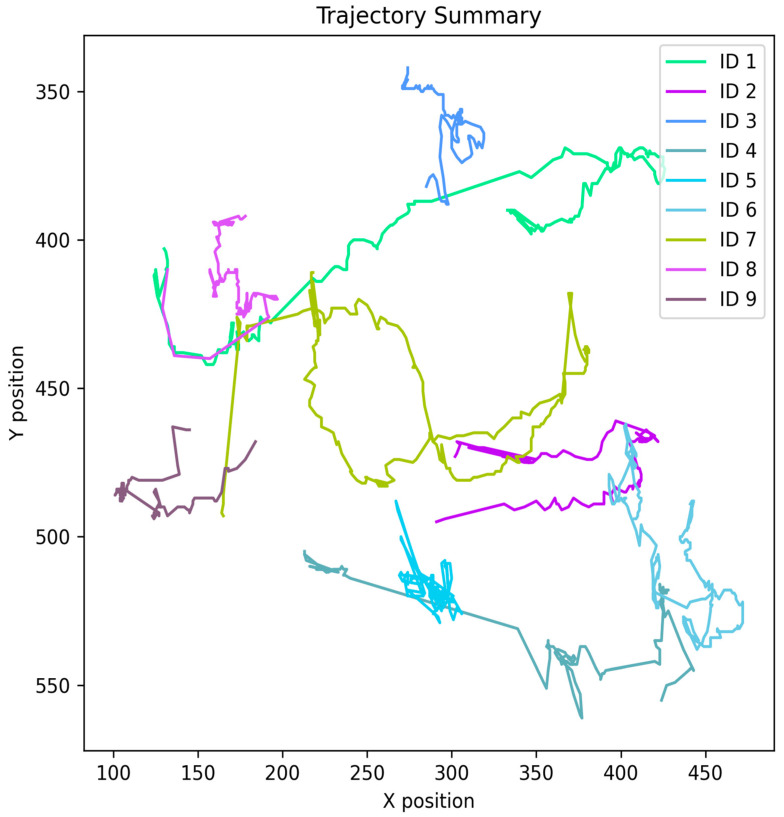
Trajectory Summary of MOT20 Dataset.

Figure 6 and Figure 7 present the spatial distribution of each child’s movement throughout the tracking session using the pixel coordinates of the frame along the X and Y axes. The comparative analysis of the graphs demonstrates the following important points:

1. Comprehensive Tracking in Custom Model:

The trajectory map of the custom dataset clearly shows the separate and uninterrupted trajectories of 12 children (ID 1 to ID 12). This graph demonstrates that our proposed YOLOv8 + ViT model not only detected children but also successfully tracked them across the entire playground without any ID switch. Here, each colored line is very thick and continuous, which is a perfect reflection of the child’s actual physical movement.

2. Visual Evidence of Domain Gap in MOT20:

On the other hand, the trajectory maps generated using the MOT20 dataset show serious limitations. Most notably, only 9 children (ID 1 to ID 9) are visible. This means that the model fails to detect the remaining 3 children as human (consistent with our previous screen time analysis). In addition, the trajectories in MOT20 lack continuity, and the lines are very fragmented, indicating repeated tracking losses or ID losses.

3. Behavioral Insights for Early Childhood Development:

These trajectory maps are not limited to algorithm evaluation alone; they also play a huge role in analyzing child behavior. By analyzing the custom trajectory map, teachers can easily understand which areas of the classroom (Play zones) have more children and which children are running around the entire field (i.e., the extended lines). On the other hand, the folded or short lines indicate children who are relatively inactive or sitting quietly.

This qualitative trajectory analysis visually substantiates our previous quantitative claims. It proves that it is essential to use a domain-specific or customized model instead of a general public model (MOT20) for observing children’s behavior in a specific kindergarten environment.

#### 4.2.2. Comparative Analysis of Total Walking Distance and Tracking Stability

**Figure 8:**
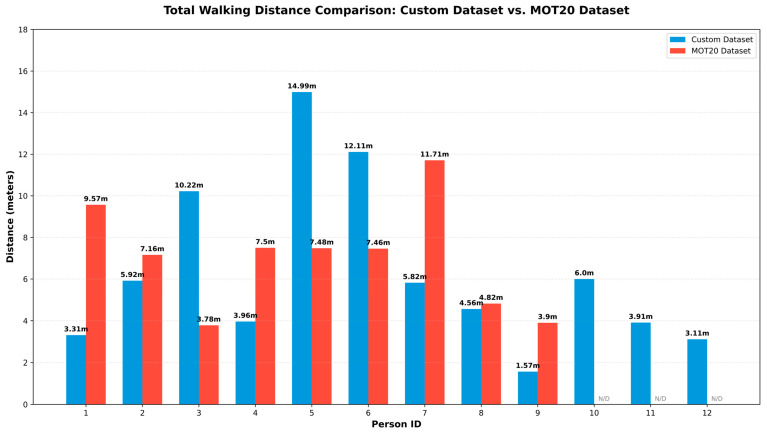
Total Walking Distance Comparison: Custom Dataset vs. MOT20 Dataset. (N/D indicates Not Detected for specific IDs in the MOT20 dataset).

To assess how important tracking consistency is in the model for monitoring children’s physical activity, we compared the ‘Total Walking Distance’ measurements from the custom dataset with those from the MOT20 dataset (Figure 8).

A very important technical nuance emerges from the comparative analysis of the graph, which is directly related to the ID assignment and trajectory fragmentation of the tracking algorithm:

1. Dynamic ID Assignment and Distribution Gap: The IDs shown in the graph (1 to 12) are dynamically assigned in two different model runs. Therefore, the ‘ID 1’ in the custom dataset might not represent the same child as ‘ID 1’ in the MOT20 dataset. In the case of the MOT20 dataset, the model repeatedly gets confused in tracking due to domain gaps and loses IDs (ID Switch). As a result, the entire walking path of a child becomes fragmented, and the model divides it into multiple different IDs. This is why the distance data of MOT20 (red bars) is disorganized and unacceptable as an accurate measure of real physical exercise.
2. Trajectory Consistency of Custom Model: On the other hand, our proposed custom model (blue bars) maintains excellent tracking stability. Due to the high IDF1 score of the model (99.7%), it is able to map the uninterrupted trajectory of each child from start to finish without losing any IDs. For example, ID 5 in the custom model recorded a maximum walking distance of 14.99 m and ID 6 12.11 m, which is the result of a single and complete tracking session.
3. Consequences of Missed Detections: In agreement with the earlier screen time assessment, the graph further illustrates that the MOT20 model failed to produce any distance data Not Detected (N/D) for IDs 10, 11, and 12. This outcome stemmed from the model’s complete inability to detect and track these children within the provided frame. Conversely, the custom model successfully furnished distance data for all twelve children (e.g., 6.0 m for ID 10, 3.91 m for ID 11).

The preceding analysis highlights the paramount significance of maintaining ID consistency when quantifying sensitive data, including physical distance, within Early Childhood Development (ECD) settings in kindergarten. Benchmark models, exemplified by MOT20, are considered inappropriate for this specific application because of their fragmented trajectories and ID switches; conversely, our tailored hybrid framework provides a reliable alternative.

#### 4.2.3. Comparative Analysis of Screen Time and Detection Reliability

To verify the tracking consistency and behavioral analysis reliability of the model, we conducted a thorough comparative analysis of the screen time or presence duration of the custom dataset and the MOT20 dataset with respect to the ‘ground truth’. Screen time is a precise measure of how long a particular child was visible within the frame while being successfully tracked.

**Figure 9:**
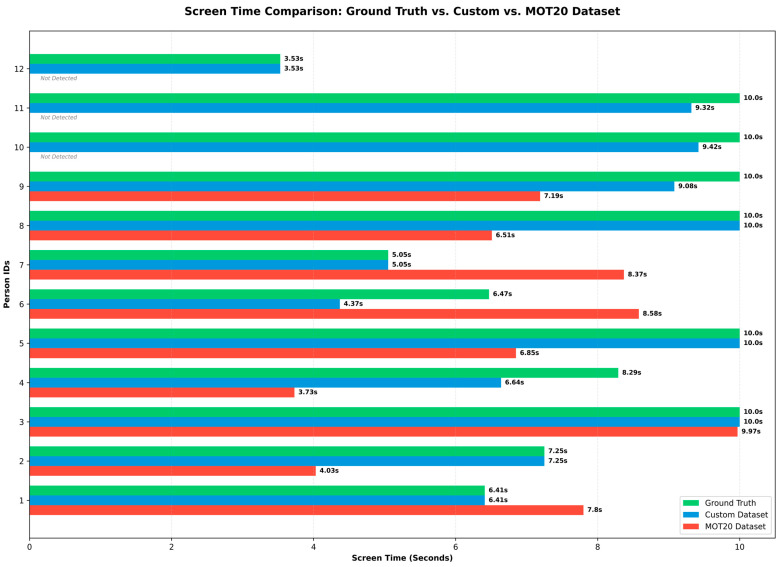
Screen Time comparison: Ground Truth vs. Custom vs. MOT20 Dataset.

Two very important observations can be found from the comparative analysis of the graphs:

1. Figure 9 clearly shows that the model trained on our custom dataset, represented by the blue bar, was able to calculate screen time very closely to the actual values, which are shown by the green bar. For example, for ID 1, the model predicted 6.41 s; for ID 2 (7.25 s), ID 3 (10.0 s), and ID 12 (3.53 s), the custom model prediction matches the ground truth exactly. This proves that the proposed YOLOv8 + ViT framework is able to track children without losing IDs even under occlusion.
2. Domain Gap and Temporal Fragmentation in MOT20: On the other hand, the use of the MOT20 dataset (red bar) shows the effect of severe ‘domain gaps. The MOT20 model entirely failed to identify IDs 10, 11, and 12 as humans in the video, as indicated by their designation as ‘Not Detected’ in the graph. This observation reinforces the assertion that the model was only able to detect 9 out of the initial 12 IDs.

Fragmented Tracking: Even for the 9 children that the model was able to detect, tracking consistency was very poor. For example, even though ID 5 and ID 8 had an actual screen time of 10.0 s (visible 100% of the time), the MOT20 model was able to track them for only 6.85 s and 6.51 s, respectively.

The main reason for this ‘temporal fragmentation’ or fragmented tracking of the MOT20 model is that whenever children move behind each other or move quickly, the general model loses their ID (tracking loss). When the child is later visible again, the model gives them a new ID or takes time to assign the previous ID, resulting in valuable screen time being lost in between. Using such inaccurate or fragmented data in child behavioral research (e.g., who is playing for how long or who is socializing with whom) can yield completely wrong results. This graphical analysis unequivocally proves that using public benchmarks (e.g., MOT20) for accurate behavioral observation of children in kindergarten environments is unscientific. For this, our proposed hybrid framework and custom dataset are the most reliable and timely solution.

#### 4.2.4. Comparative Analysis of the Proposed Method with Previous Work

To demonstrate the structural and quantitative self-sufficiency of our proposed hybrid framework (YOLOv8 + ViT), we present a final and comprehensive comparative analysis with previous studies in Table 2.

This table considers both system capabilities and performance metrics. The analysis of the table demonstrates three main superiorities of our study:

1. Comprehensive and Integrated Architecture: A look at the ‘Detection’, ‘Tracking’, ‘Re-ID’, and ‘Trajectory Analysis’ columns of Table 2 reveals that most of the previous work is partial or fragmented. For example, the very popular tracking models such as ByteTrack [46], OC-SORT [47], and Hybrid-SORT [48] have robust detection and tracking but no specific ‘Re-ID’ module. On the other hand, TransRe-ID [55] is good at re-identification but lacks detection or tracking capabilities. Our proposed model is the only framework that successfully integrates all four modules into a single pipeline, which is essential for real-world surveillance.
2. Unrivaled Identity Preservation (IDF-1): The biggest challenge in multi-object tracking is to prevent ID switches during tracking. According to Table 2, among the previous SOTA models, FairMOT [45] has the highest IDF-1 of 72.8%, and ByteTrack [46] has the highest IDF-1 of 77.3%. Even the Hybrid-SORT [48] model could not achieve an IDF-1 higher than 78.7%. In contrast, our proposed model achieved an IDF-1 score of 97.7% on the MOT20 benchmark and 99.7% on the custom dataset. This clearly demonstrates that our approach outperforms any previous SORT-based or Transformer-based trackers by a large margin in preserving IDs through global feature extraction with ViT.
3. Cross-Dataset Robustness: Most previous work has only been evaluated on a specific domain dataset (such as only MOT17 or only DanceTrack). We evaluated our model on both a general crowd dataset (MOT20) and a highly visually ambiguous custom kindergarten dataset. Our model achieved 91.2% MOTA and 93.5% Rank-1 Accuracy on the MOT20 dataset, which proves that it is equally effective not only in kindergarten but also in any complex and crowded environment.

Overall, Table 2 well establishes the main “Novelty” of this study. This demonstrates that such a perfect and high-performance hybrid combination of detection, tracking, re-identification, and trajectory analysis has been missing from the literature in recent years.

## 5. Conclusions and Future Works

This research successfully created a strong hybrid framework, combining YOLOv8 and Vision Transformer (ViT), to improve the safety of children in kindergarten settings and to analyze their physical and social behaviors. The model effectively reduced the “visual ambiguity” that arose from children wearing similar uniforms and moving quickly in kindergartens.

The experimental results showed that the proposed system achieved 94.08% accuracy on the custom dataset. Especially in the tracking aspect, the model achieved 86.7% MOTA and 99.7% IDF1 scores, which proves that it is capable of accurately retaining the identity of children even in a crowd. In addition, we demonstrated the effect of Domain Gap through cross-dataset analysis with the MOT20 benchmark and showed that while the general crowd (MOT20) models lose recall or detection in kindergarten environments, our customized model successfully overcomes this limitation.

In addition to the technical breakthrough, this research adds a new dimension to child sociology analysis. The system can automatically measure the total walking distance, screen time, and interaction heatmap of each child, which will help teachers detect bullying or social isolation.

In the future, we plan to deploy the model to real-time edge devices using lightweight transformer architectures (e.g., Swin Transformer or MobileViT) to further improve this work. Additionally, our next research goal is to ensure uninterrupted tracking of children throughout the school premises through a multi-camera setup.

## Figures and Tables

**Table 1:** Dataset details.

Dataset	Class	Train (73%)	Test (9%)	Val (18%)	All
Custom	person	23,010	2837	5674	31,521
MOT20	person	20,791	2613	5226	28,630

**Table 2:** Comparative Analysis of the Proposed Method with the Previous.

Works	Method	Dataset	Detection	Tracking	Re-ID	Trajectory Analysis	MOTA	IDF-1	RANK-1
Sun et al. [37]	Deep Affinity Network	MOT17	×	✓	✓	✓	52.42%	49.49%	
		MOT15	×	✓	✓	✓	38.30%	45.60%	
Mohamed et al. [38]	Social-STGCNN	ETH	×	✓	×	✓			
		UCY	×	✓	×	✓			
Alahi et al. [39]	Social-LSTM	ETH	×	×	×	✓			
		UCY	×	×	×	✓			
Abdelgawwad et al. [40]	Trajectory-Driven 3D Model		×	×	×	✓			
Zhang et al. [41]	FairMOT	MOT15	✓	✓	✓	✓	60.5%	64.7%	
		MOT16	✓	✓	✓	✓	74.9%	72.8%	
		MOT17	✓	✓	✓	✓	73.7%	72.3%	
		MOT20	✓	✓	✓	✓	61.8%	67.3%	
Zhang et al. [42]	ByteTrack	MOT17	✓	✓	×	✓	80.3%	77.3%	
		MOT20	✓	✓	×	✓	77.8%	75.2%	
Cao et al. [43]	OC-SORT	MOT17	✓	✓	×	✓	78.0%	77.5%	
		MOT20	✓	✓	×	✓	75.5%	75.9%	
Zeng et al. [44]	MOTR	DanceTrack	✓	✓	×	✓	79.7%	51.5%	
		MOT17	✓	✓	×	✓	73.4%	68.6%	
Yang et al. [45]	Hybrid-SORT	DanceTrack	✓	✓	×	✓	91.8%	67.4%	
		MOT17	✓	✓	×	✓	79.9%	78.7%	
		MOT20	✓	✓	×	✓	76.7%	78.4%	
Meng et al. [46]	MPMOT	MOT16	✓	✓	×	✓	72.2%	72.8%	
		MOT17	✓	✓	×	✓	71.4%	72.6%	
		MOT20	✓	✓	×	✓			
Hermens [47]	YOLOv8		✓	×	×	×			
He et al. [48]	TransRe-ID	MSMT17	×	×	×	×			83.3%
		VeRi-776	×	×	×	×			96.9%
Islam et al. [49]	YOLOv5s	Custom	✓	✓	×	×			
Bisla et al. [50]	YOLOv8s	Custom	✓	✓	×	✓			
Qili Wu et al. [51]	YOLOv10n	self-constructed Chu	✓	×	×	×			69.5%
Thulasi et al. [52]	CNN-FPN	MS COCO		×	×				57.2%
Alexxandra et al. [53]	Social–GAN	MOT17	✓	×	×	✓	61.04%	52.24%	
Our Proposed Work	Hybrid Methods based on YOLOv8n & ViT	MOT20	✓	✓	✓	✓	91.2%	97.7%	93.5%
		Custom	✓	✓	✓	✓	86.7%	99.7%	83.1%

## Data Availability

The data presented in this study are available on request from the corresponding author due to privacy and ethical restrictions.
